# Impact of Corrected Minute Ventilation on Mortality in Mechanically Ventilated Patients With COVID-19-Related Acute Respiratory Distress Syndrome: A Multicenter, Observational Study Using the J-RECOVER Registry Data

**DOI:** 10.7759/cureus.92347

**Published:** 2025-09-15

**Authors:** Jun Kataoka, Masahiro Konaka, Hiroyuki Ohbe, Koichi Hayashi, Akira Endo, Takashi Tagami, Shigeki Fujitani

**Affiliations:** 1 Department of Emergency and Critical Care Medicine, St. Marianna University School of Medicine, Kawasaki, JPN; 2 Department of Critical Care Medicine, Nerima Hikarigaoka Hospital, Tokyo, JPN; 3 Department of Clinical Epidemiology and Health Economics, School of Public Health, The University of Tokyo, Tokyo, JPN; 4 Department of Emergency and Critical Care Medicine, Tohoku University Hospital, Sendai, JPN; 5 Department of Trauma and Acute Care Surgery, Tsuchiura Kyodo General Hospital, Tsuchiura, JPN; 6 Department of Emergency and Critical Care Medicine, Jikei University School of Medicine, Tokyo, JPN

**Keywords:** acute respiratory distress syndrome (ards), covid-19, dead space, hypercapnia, invasive mechanical ventilation

## Abstract

Background

Corrected minute ventilation (VEcorr) has been proposed as a surrogate marker for dead space ventilation and may be associated with increased mortality in COVID-19-related acute respiratory distress syndrome (ARDS). However, prior studies have shown inconsistent results, and the mechanisms contributing to elevated VEcorr remain unclear.

Methodology

A multicenter, observational study was conducted using data from the J-RECOVER registry, including 335 adult patients with COVID-19-related ARDS who received invasive mechanical ventilation. VEcorr was calculated using the initial ventilator settings and arterial blood gas values. Multivariable logistic regression analysis was performed to assess the association between VEcorr and in-hospital mortality, adjusting for potential confounders.

Results

Higher VEcorr was independently associated with increased in-hospital mortality (odds ratio = 1.11; 95% confidence interval = 1.01-1.23; p = 0.039). Patients with a higher VEcorr also had higher levels of fibrin degradation products and Fibrosis-4 scores. In addition, a higher VEcorr was significantly associated with elevated PaCO_2_ (≥45 mmHg), respiratory acidosis (pH <7.25), and increased mean airway pressure (≥15 cmH_2_O). Patients with both a high VEcorr and hypercapnia had significantly higher mortality.

Conclusions

VEcorr was independently associated with mortality in mechanically ventilated COVID-19 ARDS patients and might reflect underlying microvascular pathology. Monitoring VEcorr may help identify high-risk patients and inform ventilatory and therapeutic strategies.

## Introduction

COVID-19-related acute respiratory distress syndrome (ARDS) is a major cause of critical illness, with an in-hospital mortality rate of approximately 40% [1]. Despite various advances in treatment, mortality remains high, highlighting the need for a better understanding of the unique pathophysiology of COVID-19-related ARDS, particularly its impact on ventilatory efficiency.

In patients with ARDS, microvascular injury of the alveoli occurs, and the alveolar dead space is increased. Several observational studies have shown that an increased dead space fraction (VD/VT) is an independent risk factor for mortality [2-5]. The assessment of VD/VT, however, requires the measurement of expired CO_2_, and simpler surrogates for dead space ventilation, including corrected minute volume (VEcorr) and the ventilation ratio, have been proposed and reported to be independent risk factors for mortality in ARDS patients [6-9]. Furthermore, pathological findings indicate that microthrombi in pulmonary capillaries were frequently observed in patients with COVID-19 pneumonia, suggesting that microvascular derangement increased dead space ventilation and contributed to the exacerbation of hypoxemia [10], which validates anticoagulation as part of therapeutic management [11]. It has been previously reported that VEcorr was greater in ARDS with COVID-19, compared with that due to other causes [12]. Although SARS-CoV-2 per se caused endothelial damage by binding angiotensin-converting enzyme 2 receptors [13] and showed a unique coagulopathy that differed from that caused by bacterial pneumonia [14], the factors that affect the link between deranged VEcorr and the coagulopathy in COVID-19 pneumonia have not been clarified.

Several studies have reported the relationship between increased dead space ventilation and in-hospital mortality in COVID-19 ARDS [15-17]. Fusina et al. reported that VEcorr was independently associated with in-hospital mortality in a single-center, observational study [15]. In contrast, Morales-Quinteros et al. failed to show a significant association between increased estimated dead space fraction (by the Harris-Benedict formula) and in-hospital mortality in a multicenter, observational study [16]. In this study, patients with COVID-19 ARDS exhibited a trend toward increased VD/VT in the first few days, but after adjustment for potential confounders, this trend was not independently associated with in-hospital mortality. Hence, whether increased dead space ventilation is an independent predictor of in-hospital mortality in COVID-19 ARDS remains controversial. Furthermore, the factors that are associated with increased dead space ventilation among the biomarkers for activation of coagulation or inflammation have not been fully determined.

Therefore, this study was performed to investigate whether VEcorr is associated with in-hospital mortality in COVID-19 ARDS patients, assuming VEcorr as a surrogate marker for dead space ventilation, and to elucidate the factors responsible for the elevated VEcorr in these patients.

## Materials and methods

Study design and ethics approval

This was a Japanese, multicenter, observational study including COVID-19 patients using real-world data (J-RECOVER study) [18]. The J-RECOVER study involved the construction of a database of microbiologically confirmed COVID-19 cases in Japan with the aim of promptly addressing unresolved research issues. The present study adhered to the principles of the Declaration of Helsinki and was approved by the ethics committee of each participating hospital (the Institutional Review Board at Nerima Hikarigaoka Hospital; registration number: 21040802). As this was an observational study conducted using existing medical information, the requirement for informed consent from each patient was waived. Information about the current research was disclosed to the patients and posted in the hospital or on the hospital’s website (https://nms-kosugi-eccm.com/covid19-joint-research/) to ensure that they had the opportunity to refuse to participate.

Study setting and data collection

The J-RECOVER study group called for participating facilities and submission of research proposals until the end of September 2020. Overall, 66 institutions from Japan, in which patients with moderate-to-severe COVID-19 were treated, participated voluntarily in the J-RECOVER study. Hospitalized patients with COVID-19 who had a microbiologically confirmed SARS-CoV-2 infection and were discharged from each participating institution between January 1, 2020, and September 31, 2020, were eligible for the J-RECOVER study, regardless of being admitted to the intensive care unit (ICU).

The database of the J-RECOVER study contains the following information: age at admission, sex, body mass index at admission, comorbidities at admission, Sequential Organ Failure Assessment (SOFA) hemodynamic score at admission, initial arterial blood gas measurements after the start of invasive mechanical ventilation, initial ventilator settings after the start of invasive mechanical ventilation, COVID-19 symptom onset date, date of admission, and outcome at discharge.

Study population

Using the database of the J-RECOVER study between January 1, 2020, and September 31, 2020, hospitalized COVID-19 patients were identified who met the following inclusion criteria: (i) 18 years or older, (ii) received invasive mechanical ventilation during hospitalization, (iii) had ARDS defined by the Berlin Definition (PaO_2_/FiO_2_ ratio ≤300) at the initial arterial blood gas analysis after the start of invasive mechanical ventilation, and (iv) had initial ventilator settings and initial arterial blood gas results after the start of invasive mechanical ventilation available. Patients who were intubated before hospitalization and were transferred from another hospital were excluded.

Study outcome

The primary outcome in this study was in-hospital mortality.

Exposure

The main exposure of interest in this study was VEcorr [8]. Following the previous literature, VEcorr was calculated as minute ventilation multiplied by the actual PaCO_2_ at the time of initial arterial blood gas analysis after invasive mechanical ventilation, divided by the ideal PaCO_2_ value of 40 mmHg.

Statistical analysis

VEcorr and the patients’ characteristics are presented overall and stratified by survivors and non-survivors. An unadjusted analysis comparing survivors and non-survivors was performed using the chi-squared test for binary variables and the Wilcoxon rank-sum test for continuous variables.

To assess the linear association between VEcorr at the time of initial arterial blood gas analysis after the start of invasive mechanical ventilation and in-hospital mortality, a multivariable logistic regression analysis was performed with in-hospital mortality as a dependent variable, with continuous VEcorr and other parameters pertaining to patient characteristics as independent variables. As there were some missing data (i.e., body mass index (BMI) (n = 27), SOFA score (n = 68), lactate (n = 6), length from COVID-19 symptom onset to hospitalization (n = 3), and mean airway pressure (n = 122)), the missing data were assumed to occur at random, and multiple imputations were performed by chained equations [19], creating 20 imputed datasets with the primary outcome, VEcorr, and all patient characteristics. The “mi impute chained” command in STATA was used [20]. Imputation estimates and standard errors were obtained based on Rubin’s rule [19].

A logistic regression analysis using quartile categories of VEcorr instead of VEcorr as a continuous variable was also performed. In addition, similar analyses were performed with age, Krebs von den Lungen-6 (KL-6), fibrin degradation products (FDPs), D-dimer, and the Fibrosis-4 (FIB-4) score as quartile categories to evaluate their associations with in-hospital mortality. The FIB-4 score, calculated from age, aspartate aminotransferase, alanine aminotransferase, and platelet count, was originally proposed as a non-invasive marker of hepatic fibrosis [21] and has recently been accepted as an indirect marker representing systemic stress and the inflammatory response in COVID-19 [22]. Based on these considerations, the FIB-4 score was included as a variable in the analysis.

The areas under the receiver operating characteristic (ROC) curves (AUCs), sensitivity, specificity, and positive and negative predictive values of VEcorr for in-hospital mortality were calculated. The Youden index was used to determine the optimal cut-off point. With this cut-off point, clinical parameters in patients in the high and low VEcorr groups were compared to investigate factors associated with elevated VEcorr, with a particular focus on markers related to coagulation, organ injury, and pulmonary function. A multivariable logistic regression analysis was also conducted to explore the association between these factors and high VEcorr. Finally, to assess the relationship with mechanical ventilation strategies, the distributions of PaCO_2_ and mean airway pressure were plotted for patients with high and low VEcorr, and their associations with in-hospital mortality were examined.

Continuous variables are presented as median with interquartile range (IQR) values, and categorical variables are presented as counts with percentages. All reported p-values are two-sided, and differences with a p-value <0.05 were considered significant. All analyses were performed using STATA/SE 17.0 software (StataCorp, College Station, TX, USA) or the John Macintosh Project (JMP) statistical software (version 17, SAS Institute Inc., Cary, NC, USA).

## Results

Patients’ characteristics

**Figure 1:**
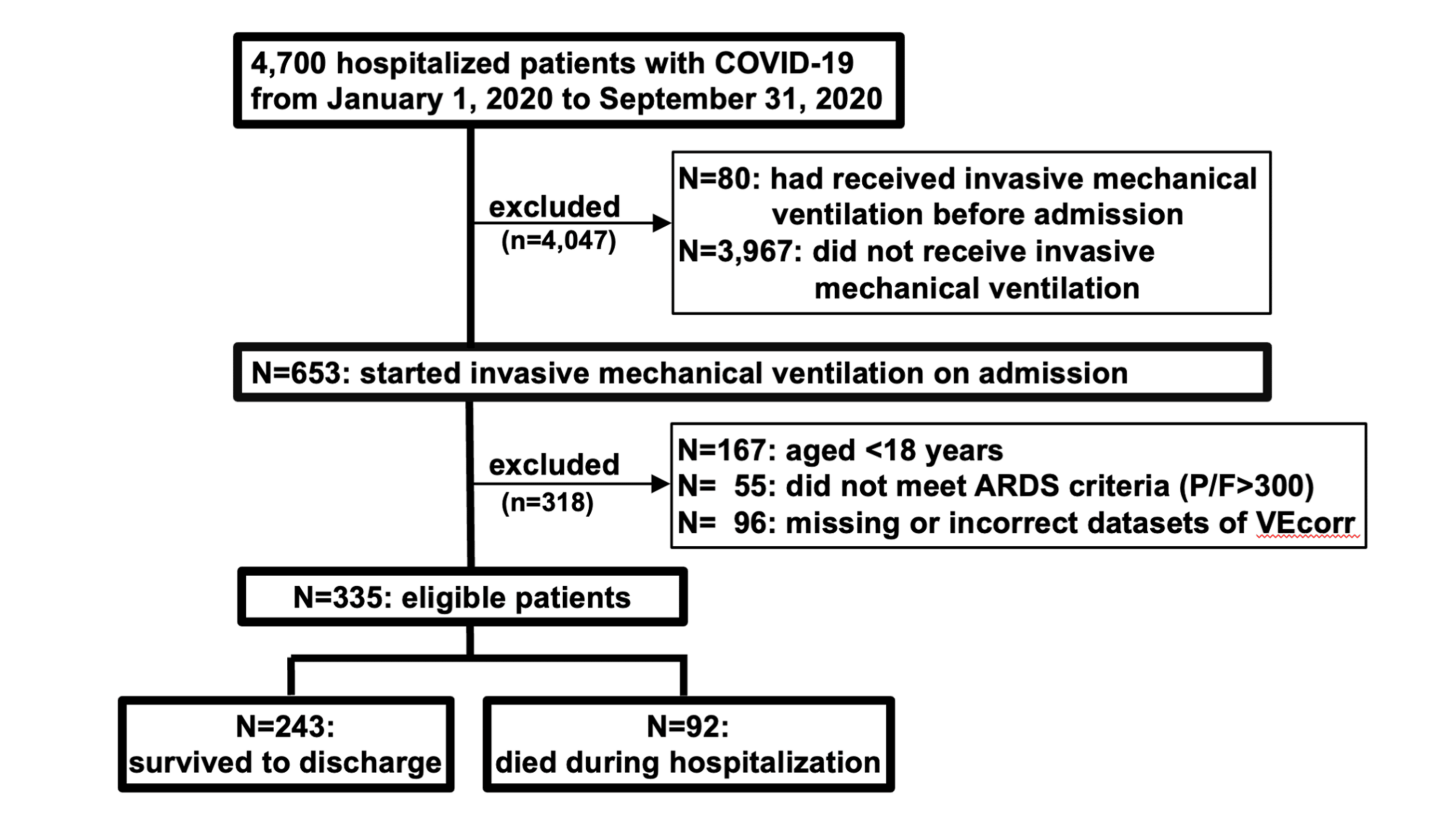
Patient enrollment. VEcorr: corrected minute ventilation

A total of 335 patients were eligible for this study (Figure 1). Their median age was 68 years, and 77.6% were male (Table 1). The median SOFA score at admission was 3 (IQR = 2-6). The median PaO_2_/FiO_2_ ratio at the initial arterial blood gas analysis after the start of invasive mechanical ventilation was 161 (IQR = 125-208). The median VEcorr at the initial arterial blood gas analysis after the start of invasive mechanical ventilation was 7.9 (IQR = 6.3-10.0). The overall in-hospital mortality was 27.5% (n = 92/335). Unadjusted analyses showed that survivor patients were younger and had lower VEcorr at the initial arterial blood gas analysis after ventilation, lower SOFA score at admission, longer length from COVID-19 symptom onset to hospitalization, lower D-dimer, lower KL-6, and lower FIB-4 score than non-survivor patients.

**Table 1:** Patients’ baseline characteristics. BMI: body mass index; SOFA: Sequential Organ Failure Assessment; MV: mechanical ventilation; VEcorr: corrected minute ventilation; FDP: fibrin degradation product; KL-6: Krebs von den Lungen-6; FIB-4: Fibrosis-4; IQR: interquartile range

	Total	Survivors	Non-survivors	P-value
	N = 335	N = 243	N = 92	
Age, years, median (IQR)	68 (57-76)	64 (53-73)	76 (69.5-81)	<0.001
Male, n (%)	260 (77.6%)	193 (79.4%)	67 (72.8%)	0.20
BMI (kg/m^2^), median (IQR)	25.0 (22.2-28.2)	25.3 (22.4-28.4)	24.2 (22.0-27.6)	0.13
Diabetes mellitus, n (%)	92 (27.5%)	70 (28.8%)	22 (23.9%)	0.37
Hypertension, n (%)	51 (15.2%)	37 (15.2%)	14 (15.2%)	1.00
SOFA score at admission, median (IQR)	3 (2-6)	3 (2-6)	4 (3-7)	0.020
Lactate (mmol/L), median (IQR)	1.1 (0.9-1.5)	1.1 (0.8-1.5)	1.1 (0.9-1.6)	0.19
Length from COVID-19 symptom onset to hospitalization, median (IQR)	8 (5-10.5)	8 (6-11)	7 (4-10)	0.007
Length from hospitalization to MV, median (IQR)	1 (1-3)	1 (1-3)	1 (1-3)	0.75
PaO_2_/FiO_2_ ratio_first, median (IQR)	161 (125-208)	164 (129-212)	147 (120-201)	0.170
Mean airway pressure, median (IQR)	15 (12-16.8)	15 (12-16)	15 (12.8-18)	0.24
VEcorr_first, median (IQR)	7.9 (6.3-10.0)	7.7 (6.1-9.9)	8.5 (6.9-11.0)	0.019
FDP (µg/mL), median (IQR)	5.2 (3.3-9.3)	5.2 (3.5-8.8)	6.85 (3.88-15.7)	0.097
D-dimer (µg/mL), median (IQR)	1.6 (1.0-3.3)	1.5 (0.9-2.8)	2.6 (1.3-5.9)	0.011
KL-6 (U/mL), median (IQR)	365 (244-579)	339 (239-513)	540 (259-861)	0.004
FIB-4 score, median (IQR)	3.0 (1.8-4.7)	2.6 (1.6-4.2)	4.4 (3.32-6.7)	<0.001
Medications, n (%)				
Heparin	217 (64.8%)	142 (58.4%)	75 (81.5%)	<0.001
Aspirin	33 (9.9%)	18 (7.4%)	15 (16.3%)	0.015
Statins	42 (12.5%)	28 (11.5%)	14 (15.2%)	0.362

Exploration of factors affecting patients’ mortality

**Figure 2:**
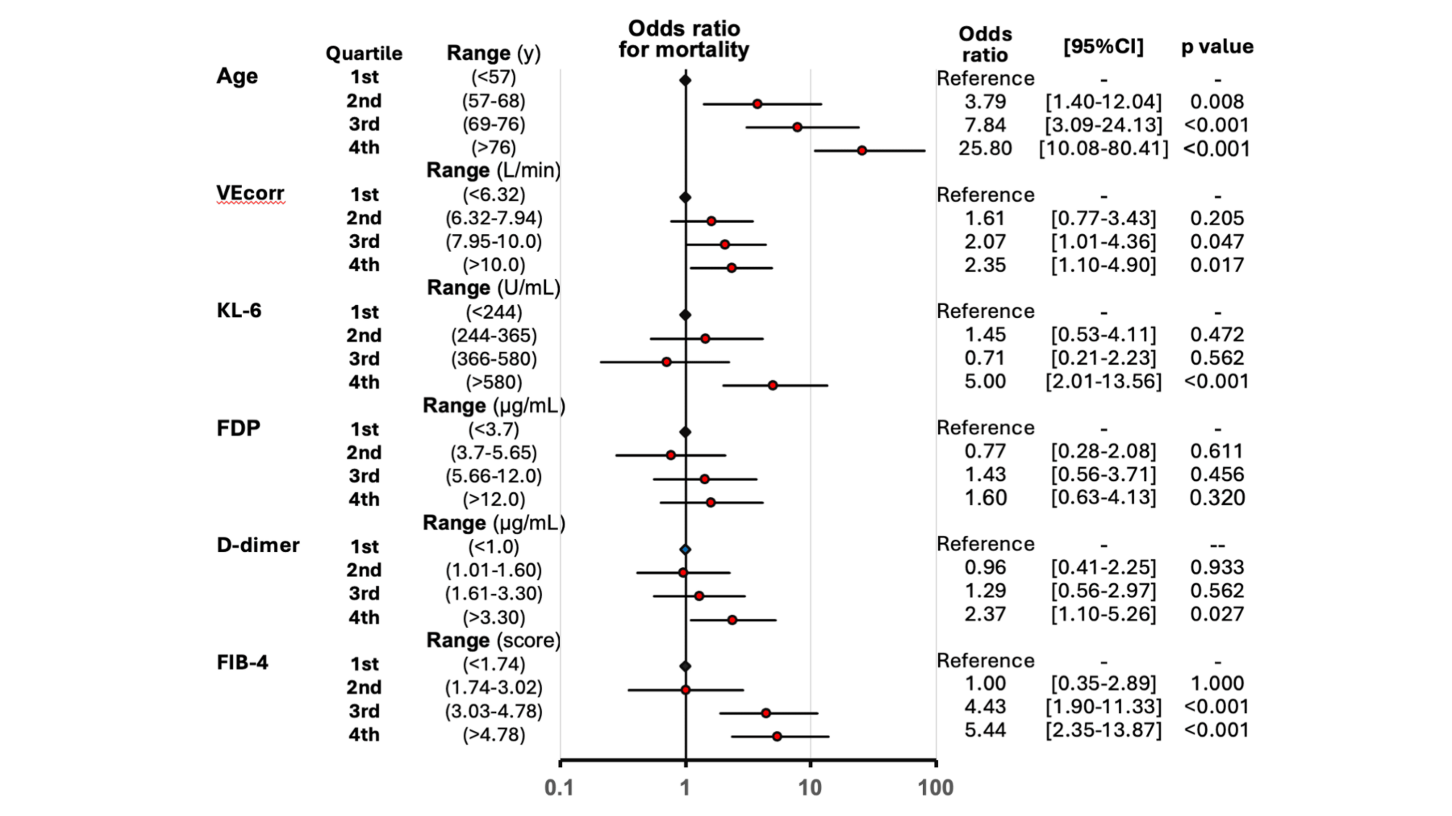
Odds ratios for mortality stratified by quartiles for each parameter. VEcorr: corrected minute ventilation; FDP: fibrin degradation product; KL-6: Krebs von den Lungen-6; FIB-4: Fibrosis-4; CI: confidence interval

The multivariable logistic regression analysis showed that higher VEcorr as a continuous variable was significantly associated with higher in-hospital mortality (odds ratio (OR) = 1.11, 95% confidence interval (95% CI) = 1.01 to 1.23), p = 0.039) (Table 2). Furthermore, higher age, higher SOFA score at admission, shorter length from COVID-19 symptom onset to hospitalization, and higher mean airway pressure at initial ventilation settings after the start of invasive mechanical ventilation were associated with higher in-hospital mortality. Logistic regression analysis with quartile categories of VEcorr also showed that the third and the fourth quartiles of VEcorr were significantly associated with higher in-hospital mortality, using the first quartile of VEcorr as the reference (Figure 2). Similarly, the second, third, and fourth quartiles of age, the fourth quartiles of KL-6 and D-dimer, and the third and fourth quartiles of the FIB-4 score were significantly associated with higher in-hospital mortality.

**Table 2:** Odds ratios for hospital mortality after multiple imputation. VEcorr: corrected minute ventilation; SOFA: Sequential Organ Failure Assessment; MV: mechanical ventilation; CI: confidence interval

	Odds ratio (95% CIs)	P-value
VEcorr_first	1.11 (1.01-1.23)	0.039
Age	1.12 (1.08-1.16)	<0.001
Male	1.07 (0.52-2.19)	0.857
Body mass index	1.00 (0.96-1.05)	0.933
Diabetes mellitus	0.83 (0.42-1.63)	0.581
Hypertension	0.80 (0.34-1.88)	0.606
SOFA score at admission	1.18 (1.01-1.38)	0.043
Lactate	1.05 (0.79-1.38)	0.756
Length from COVID-19 symptom onset to hospitalization	0.92 (0.85-1.00)	0.038
Length from hospitalization to MV	1.07 (0.99-1.16)	0.083
Mean airway pressure	1.12 (1.03-1.23)	0.013
PaO_2_/FiO_2_ ratio_first	0.83 (0.51-1.33)	0.432

Factors affecting VEcorr

**Figure 3:**
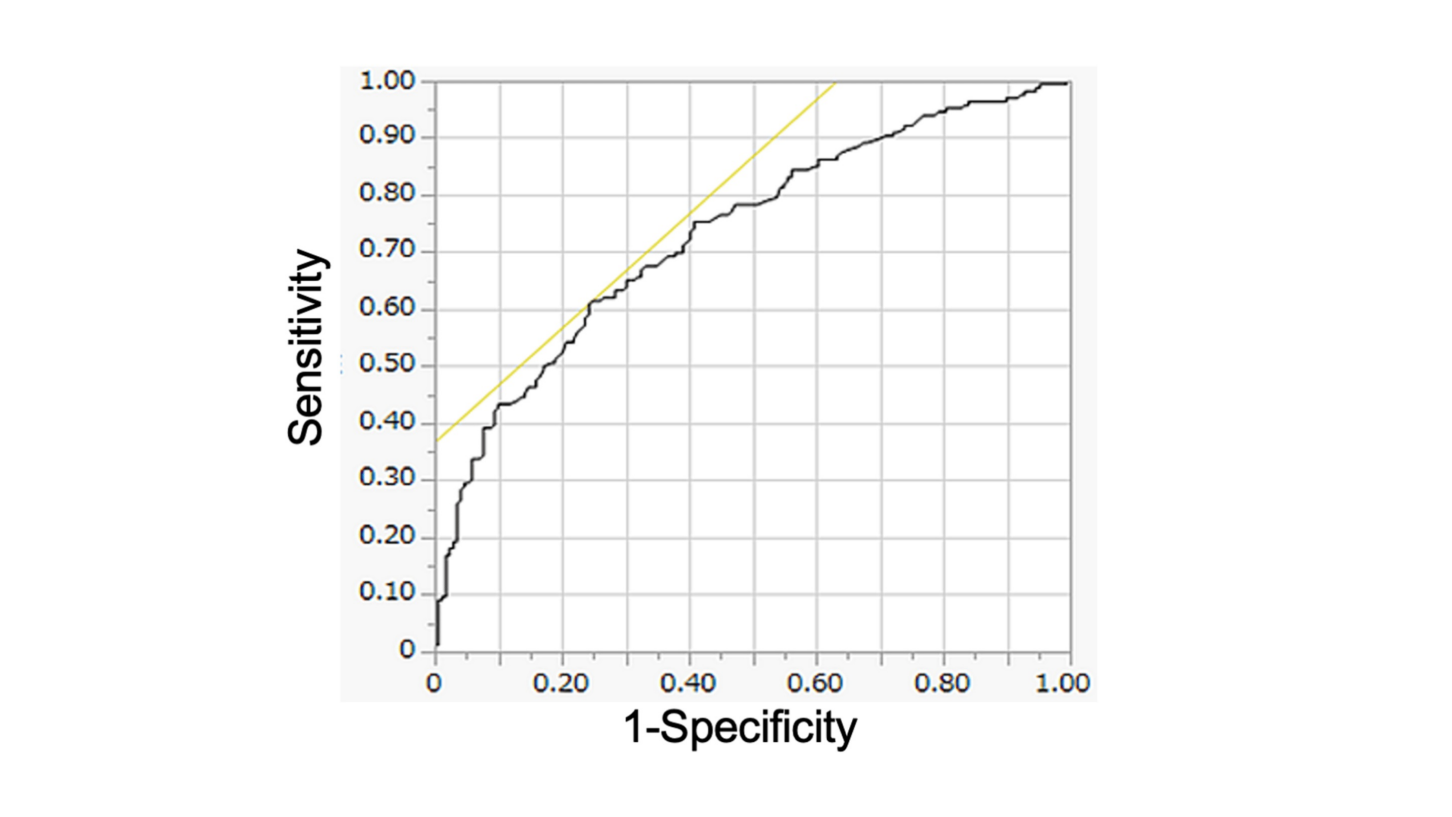
ROC curve to determine the optimal cut-off value for VEcorr predicting patients’ mortality. AUC: 0.733; cut-off of VEcorr: 8.0; sensitivity: 0.615; specificity: 0.751. ROC: receiver operating characteristic; AUC: area under the curve; VEcorr: corrected minute ventilation

The ROC analysis for predicting in-hospital mortality identified an optimal cut-off value of 8.0 L/minute for VEcorr (Figure 3). Using this value, the patients were dichotomized, and it was found that the patients with higher VEcorr (≥ 8.0 L/minute) were more likely to be male and have higher values of hematocrit, lactate, PaCO_2_, mean airway pressure, and lactate dehydrogenase (LDH) than those with lower VEcorr (<8.0 L/minute) (Table 3).

**Table 3:** Parameters stratified by VEcorr. VEcorr: corrected minute ventilation; BMI: body mass index; CRP: C-reactive protein; LDH: lactate dehydrogenase; FDP: fibrin degradation product; KL-6: Krebs von den Lungen-6; FIB-4: Fibrosis-4; IQR: interquartile range

	VEcorr <8.0	VEcorr ≥8.0	P-value
	(N = 169)	(N = 166)	
Age, years, median (IQR)	69 (57.5-76.5)	67 (56-74)	0.140
Male, n (%)	119 (70.4%)	141 (84.9%)	0.001
BMI (kg/m^2^), median (IQR)	25.2 (22.0-27.7)	24.9 (22.3-28.6)	0.472
Hematocrit (%), median (IQR)	37.5 (33.2-41.7)	39.7 (36.1-43.5)	0.004
Platelets (10^4^/uL), median (IQR)	18.1 (14.4-24.5)	17.8 (13.7-24.4)	0.883
Lactate (mmol/L), median (IQR)	1.1 (0.8-1.5)	1.3 (0.9-1.7)	0.018
PaO_2_ (mmHg), median (IQR)	80.8 (66.0-110.0)	73.0 (59.5-97.9)	0.106
PaCO_2_ (mmHg), median (IQR)	40 (36-44)	46 (41-54)	<0.001
Mean airway pressure (cmH_2_O), median (IQR)	14 (12-16)	15 (13-18)	<0.001
CRP (mg/dL), median (IQR)	11.5 (5.2-17.2)	11.5 (6.4-16.7)	0.746
LDH (IU/L), median (IQR)	406 (312-503)	444 (354-569)	0.011
FDP (µg/mL), median (IQR)	5.6 (3.7-9.1)	5.5 (3.5-13.2)	0.620
D-dimer (µg/mL), median (IQR)	1.7 (1.0-3.3)	1.5 (0.9-3.5)	0.529
Fibrinogen (mg/dL), median (IQR)	539 (458-614)	553 (465-659)	0.333
KL-6 (U/mL), median (IQR)	347 (236-542)	400 (250-650)	0.203
FIB-4 score, median (IQR)	2.95 (1.77-4.57)	3.21 (1.66-5.15)	0.549
Medications, n (%)			
Heparin, n (%)	115 (68.1%)	102 (61.5%)	0.206
Statin, n (%)	25 (14.8%)	17 (10.2%)	0.208

**Figure 4:**
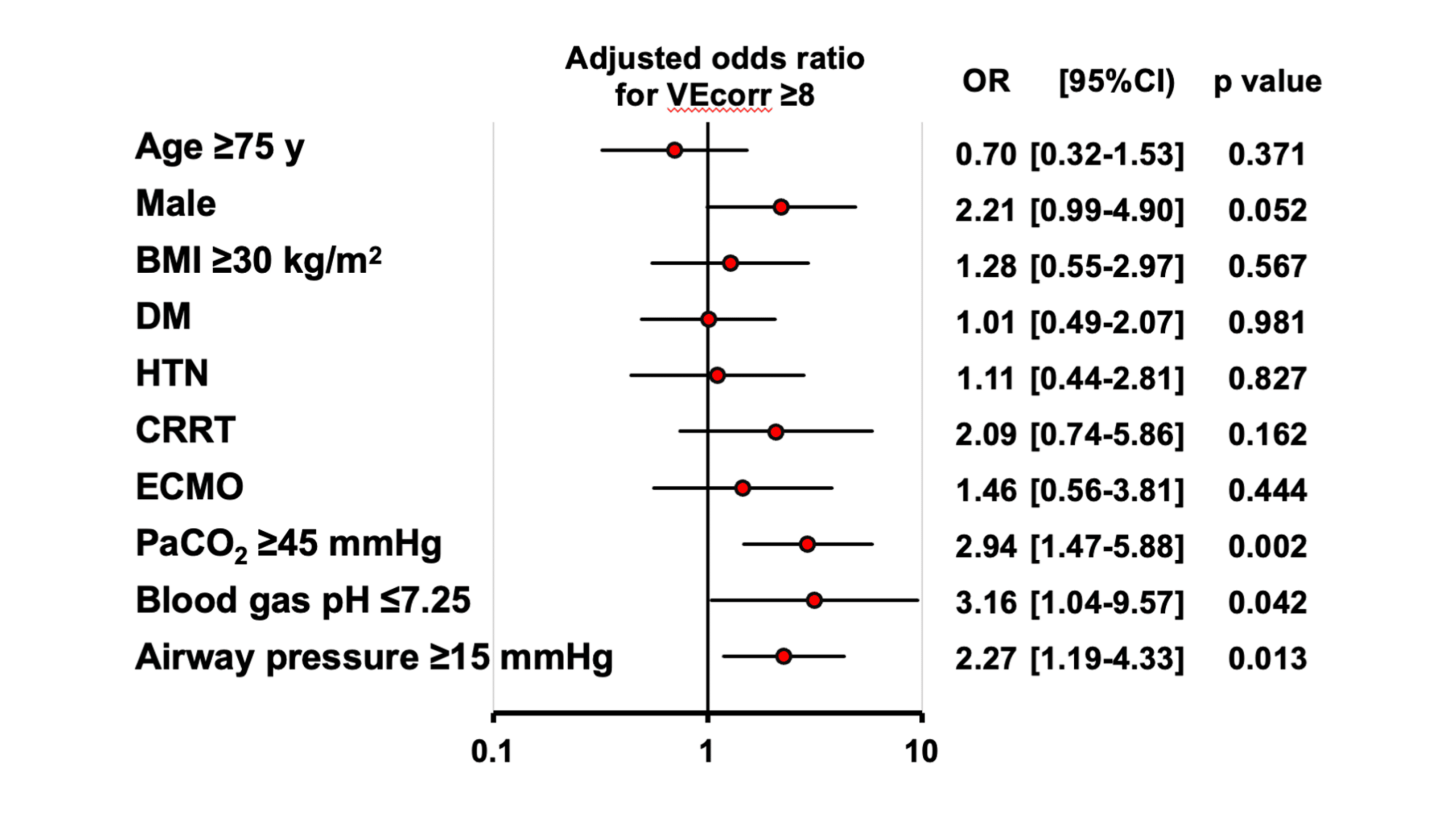
Impacts of various factors associated with pulmonary function on VEcorr VEcorr: corrected minute ventilation; DM: diabetes mellitus; HTN: hypertension; CRRT: continuous renal replacement therapy; ECMO: extracorporeal membrane oxygenation

**Figure 5:**
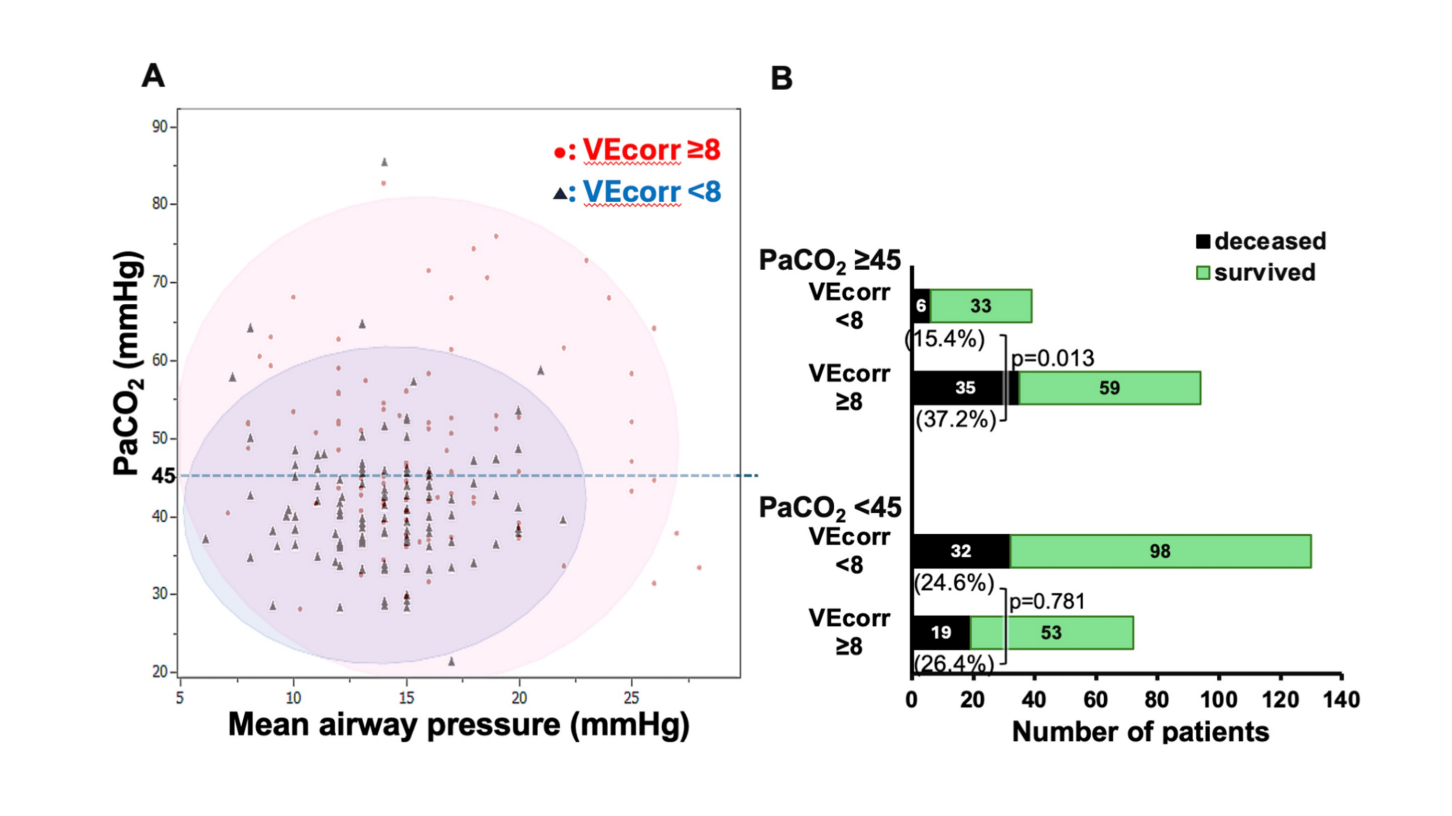
Distribution of VEcorr and mortality. (A) Association between VEcorr and PaCO_2_/mean airway pressure distribution. Red circles represent individual patients with VEcorr ≥8 L/minute, and black triangles represent those with VEcorr <8 L/minute, plotting PaCO_2_ versus mean airway pressure. Colored circles indicate 95% confidence intervals for each VEcorr group. (B) Mortality. The upper panel shows bar charts of the number of survivors and non-survivors among patients with PaCO_2_ ≥45 mmHg, stratified by VEcorr (<8 vs. ≥8 L/minute; mortality 15.4% vs. 37.2%, p = 0.013). The lower panel shows bar charts of the number of survivors and non-survivors among patients with PaCO_2_ <45 mmHg, stratified by VEcorr (<8 vs. ≥8 L/minute; mortality 24.6% vs. 26.4%, p = 0.781). VEcorr: corrected minute ventilation

**Figure 6:**
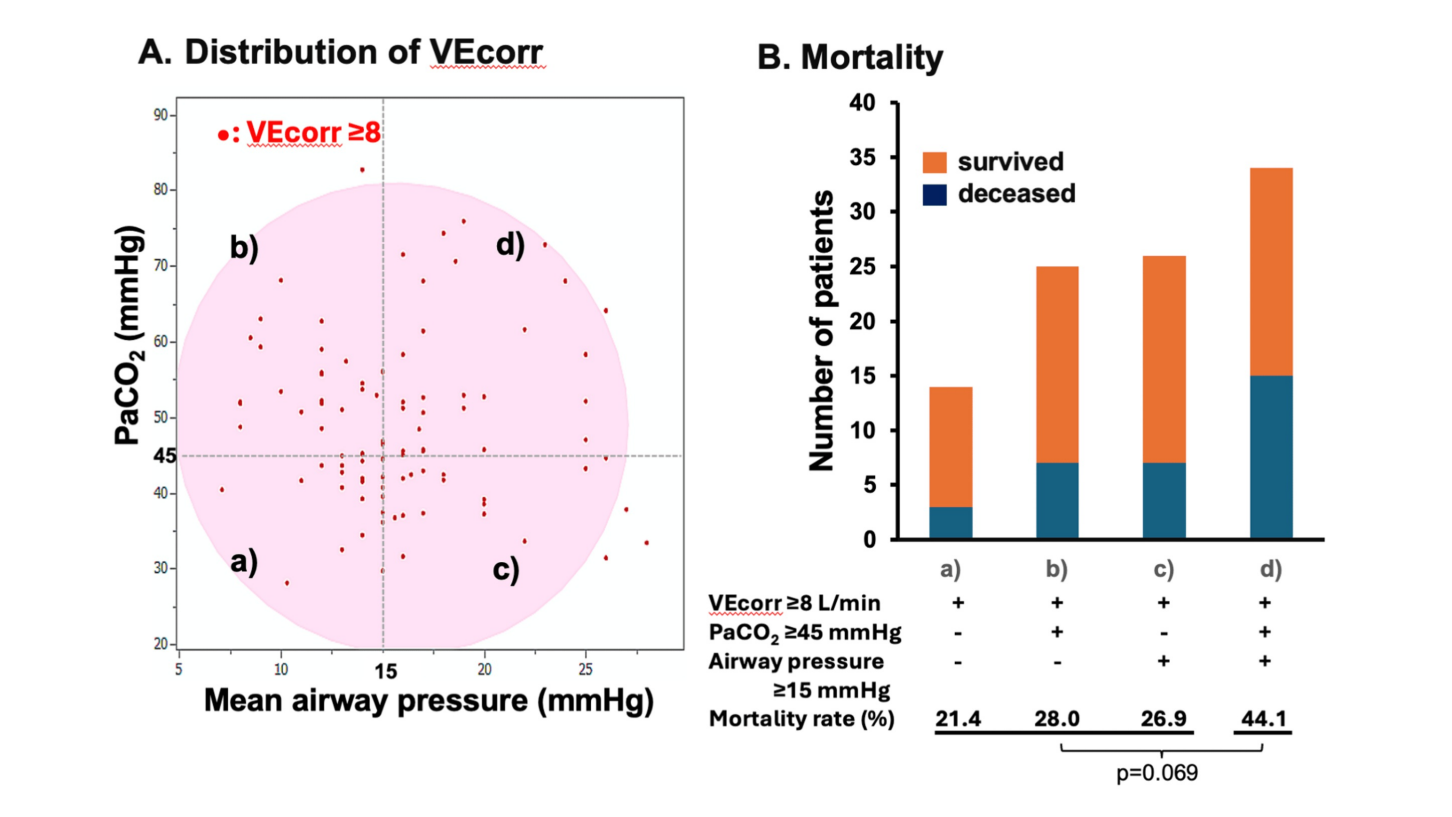
PaCO2/Mean airway pressure distribution in patients with VEcorr ≥8 L/minute and mortality. (A) Distribution of VEcorr. Colored circles indicate 95% confidence intervals. Patients with VEcorr ≥8 L/minute were stratified into four groups according to PaCO₂ (cut-off of 45 mmHg) and mean airway pressure (cutoff of 15 cmH₂O): (a) PaCO₂ <45 mmHg and mean airway pressure <15 cmH₂O, (b) PaCO₂ ≥45 mmHg and mean airway pressure <15 cmH₂O, (c) PaCO₂ <45 mmHg and mean airway pressure ≥15 cmH₂O, and (d) PaCO₂ ≥45 mmHg and mean airway pressure ≥15 cmH₂O. (B) Mortality. Mortality rates among the four groups are shown. Group (d) exhibited a higher mortality rate (44.1%) compared with groups (a), (b), and (c); however, the difference did not reach statistical significance (p = 0.069). VEcorr: corrected minute ventilation

Regarding the relationship between lung function and VEcorr, higher PaCO_2_ (≥45 mmHg), acidemia (pH <7.25), and elevated mean airway pressure (≥15 mmHg) were significantly associated with higher VEcorr (≥8.0 L/minute) (Figure 4). In addition, the association between VEcorr and mechanical ventilation strategies was assessed (Figure 4). Patients with lower VEcorr (<8.0 L/minute) generally had well-controlled PaCO_2_, whereas those with higher VEcorr (≥8.0 L/minute) often had PaCO_2_ ≥45 mmHg despite higher mean airway pressure (Figures 5A, 6A). Furthermore, in the group of patients with a PaCO_2_ ≥45 mmHg, patients with a higher VEcorr (≥ 8.0 L/minute) had a significantly higher mortality rate than those with a lower PaCO_2_ (<8.0 L/minute, p = 0.013) (Figure 5B). This trend was not observed in patients with PaCO_2_ <45 mmHg (p = 0.781). In addition, when patients were stratified into four groups based on a cutoff of 15 mmHg for mean airway pressure and 45 mmHg for PaCO_2_, the group with both elevated PaCO_2_ and elevated mean airway pressure showed the highest mortality rate of all the groups (Figure 6B).

Blood coagulation, FIB-4 score, and VEcorr in COVID-19 ARDS

The association between VEcorr and the factors related to blood coagulation and organ injury was evaluated in patients with PaCO_2_ ≥45 mmHg. The increases in FDP and the FIB-4 score were significantly associated with a higher probability of elevated VEcorr (≥ 8.0 L/minute), with ORs of 1.13 (95% CI = 1.00-1.37) per every 1 µg/mL increment (p = 0.050) and 1.89 (95% CI = 1.14-4.06) per 1 score unit (p = 0.009), respectively (Table 4).

**Table 4:** Impacts of various factors associated with coagulation and organ injury on VEcorr in patients with PaCO2 ≥45 mmHg. VEcorr: corrected minute ventilation; KL-6: Krebs von den Lungen-6; FDP: fibrin degradation product; FIB-4: Fibrosis-4; CI: confidence interval

	Unit increment	Adjusted odds ratio	95% CI	P-value
Hematocrit	Every 1%	1.13	0.93-1.42	0.212
Platelets	Every 10^4^/µL	1.00	0.98-1.02	0.952
Albumin	Every 1 g/dL	0.64	0.20-1.61	0.331
Fibrinogen	Every 100 mg/mL	1.14	0.61-2.23	0.691
KL-6	Every 100 U/mL	0.94	0.54-1.72	0.841
FDP	Every 1 µg/mL	1.13	1.00-1.37	0.050
D-dimer	Every 1 µg/mL	0.95	0.87-1.02	0.201
FIB-4 score	Every 1 score	1.89	1.14-4.08	0.009

## Discussion

Establishing the therapeutic strategy for ARDS constitutes one of the most critical determinants of the patients’ survival, of which increased dead space ventilation is an independent risk factor for mortality [1-4]. In ARDS patients with COVID-19, however, the association between dead space ventilation and mortality remains inconclusive, with divergent results reported in previous studies [15,16]. Furthermore, the factors that affect dead space in ARDS patients with COVID-19, in whom microthrombi and coagulopathy are prevalent in the alveolar and systemic microcirculation, have not been determined [10,23].

The present study, a multicenter, observational analysis using a large national database, was conducted to explore the role of dead space ventilation in COVID-19 ARDS patients using VEcorr as a surrogate marker; it found that VEcorr was significantly higher in non-survivors (Table 1). Furthermore, multivariable logistic regression analysis incorporating multiple imputation demonstrated that mortality risk increased with rising VEcorr (OR of 1.11 per unit increase, 95% CI = 1.01-1.23, p = 0.039) (Table 2). Finally, stratification by VEcorr quartiles showed a clear association between higher VEcorr and mortality (Figure 2). As the association paralleled that observed with age stratification (Figure 2), which indicated a strong association with mortality, this raised the possibility that age could partially mediate the relationship between VEcorr and mortality. Therefore, an age-stratified analysis was performed, which confirmed that, even within age subgroups, elevated VEcorr (≥8 L/minute) was associated with higher mortality, though some inconsistencies were noted, particularly at age ≤68 years (Figure 3). These results may help explain why previous studies reported conflicting findings [14,15] and suggest that the effect of VEcorr on mortality could vary across different age groups or populations.

In addition to the relationship between VEcorr and mortality, the present study explored the factors associated with elevated VEcorr. Elevated LDH was found to be higher in patients with higher VEcorr (≥8 L/minute) (Table 3). Furthermore, FDP and the FIB-4 score were significantly associated with higher VEcorr (Table 4), suggesting that pulmonary microvascular thrombosis and systemic endothelial injury contributed to increased dead space ventilation in COVID-19 ARDS. Furthermore, LDH may serve as a valuable biomarker reflecting tissue injury and hypoxia secondary to pulmonary microvascular inflammation and microthrombus formation in COVID-19-associated respiratory failure [24]. Notably, the association between elevated FDP and high VEcorr suggests that the formation of pulmonary microthrombi may be a mechanism contributing to increased dead space ventilation [23,25]. In contrast, in a retrospective, observational study involving patients with COVID-19 ARDS, no significant association was observed between D-dimer and VEcorr [26]. Similarly, the present study failed to demonstrate a clear relationship between VEcorr and D-dimer, but, instead, showed a significant association between VEcorr and FDP. This finding suggests that FDP, rather than D-dimer, could serve as a more sensitive indicator of microvascular thrombosis contributing to increased dead space ventilation in COVID-19-related ARDS. The therapeutic effect of heparin administration could not be evaluated in the present study, as detailed information regarding its timing and dosage was unavailable.

The FIB-4 score, originally developed as a non-invasive marker to assess liver fibrosis [21], has recently attracted attention in the context of COVID-19 as a potential prognostic biomarker [22,27]. Although the FIB-4 score primarily reflects liver injury and fibrosis, its elevation in COVID-19 may indicate broader systemic endothelial and microvascular dysfunction. An elevated FIB-4 score was found to be associated with increased VEcorr (Table 4), a surrogate marker of dead space ventilation, suggesting a potential link between hepatic dysfunction and impaired pulmonary microcirculation. Moreover, many studies have shown that platelet aggregation and coagulopathy play a central role in the pathogenesis of COVID-19-related organ damage [23,25,28]. The present findings demonstrating the relationships of the FIB-4 score and FDP with increased VEcorr further support the hypothesis that the FIB-4 score may serve as a surrogate marker not only of hepatic injury but also of microvascular damage in COVID-19.

From a physiological standpoint, increased dead space ventilation decreases ventilatory efficiency, resulting in the retention of CO_2_ and a concomitant decrease in blood pH (respiratory acidosis). In clinical practice, in an effort to compensate for this inefficiency and to normalize PaCO_2_, clinicians often adjust ventilator settings (e.g., increasing respiratory rate and tidal volume), which can inadvertently lead to higher mean airway pressure. The present findings thus support this pathophysiological relationship; high VEcorr not only increased PaCO_2_ but also caused higher mean airway pressure (Figure 3). Importantly, in the subgroup of patients with PaCO_2_ ≥45 mmHg, those with higher VEcorr had a higher mortality rate (Figure 4B), suggesting a detrimental role of permissive hypercapnia in the setting of increased dead space. This observation calls into question the safety of permissive hypercapnia in patients with severely compromised ventilatory efficiency and underlines the importance of individualized ventilation strategies. In this regard, a multicenter, observational study by Nin et al. [9] showed that elevated PaCO_2_ was independently associated with higher ICU mortality in patients with moderate-to-severe ARDS, even after adjusting for ventilator parameters and disease severity. Moreover, in a recent, retrospective, cohort study, severe hypercapnia (PaCO_2_ ≥ 50 mmHg) during the early phase of ARDS was associated with higher ICU mortality, particularly when hypercapnia persisted over several days [29]. Finally, a systematic review and meta-analysis in patients with ARDS showed that, though permissive hypercapnia might be protective in some settings, imposed hypercapnia due to impaired ventilatory efficiency was associated with worse clinical outcomes [30]. In concert, whereas mild hypercapnia may be acceptable in some patients, persistent or marked hypercapnia, particularly in the context of poor ventilatory efficiency, should be approached with caution. Early recognition of this high-risk phenotype may warrant consideration of alternative ventilation strategies, including extracorporeal support. Prolonged exposure to high airway pressure and hypercapnia in this population could lead to worse outcomes unless addressed in a timely manner.

Finally, this study has limitations inherent to its retrospective design. First, detailed ventilator settings were not available, particularly plateau pressure and driving pressure, making it impossible to calculate respiratory system compliance. Second, VEcorr is a surrogate marker for dead space ventilation; though more accurate measures such as VD/VT would have been preferred, they were unavailable due to significant missing data. Third, only initial ventilator settings and arterial blood gas measurements were analyzed, and changes in ventilation parameters or disease trajectory over time were not evaluated. Thus, the influence of subsequent ventilator management strategies on patient outcomes could not be fully captured. Moreover, as this was an observational study, the design was not intended to evaluate the effects of specific ventilatory interventions. To address potential biases from missing data, multiple imputation techniques were applied for key variables in the multivariable analysis. Importantly, the results from complete case analyses and those incorporating multiple imputation were consistent, providing internal validity and acting as a form of sensitivity analysis. Fourth, this study has generalizability constraints. The data were collected between January and September 2020, before the emergence of later SARS-CoV-2 variants and the widespread adoption of treatments such as corticosteroids, antiviral agents, and vaccination. Additionally, as the study cohort was limited to Japanese patients, the findings may not be directly applicable to populations with different ethnic, demographic, or clinical characteristics. Fifth, although several biomarkers, including FDP, KL-6, and FIB-4, were analyzed, assay methods were not standardized across the 66 participating hospitals, potentially introducing inter-laboratory variability that could affect the accuracy and comparability of the measurements.

## Conclusions

In this multicenter study of patients with COVID-19-related ARDS, elevated VEcorr, a surrogate marker for dead space ventilation, was independently associated with in-hospital mortality. This association appears to be linked to microvascular injury and thrombosis, as reflected by elevated FDP and FIB-4 score. Moreover, increased PaCO_2_ in patients with higher VEcorr further exacerbates the risk of poor outcomes. These findings highlight the pathophysiological importance of dead space ventilation in COVID-19 ARDS and suggest the need for careful consideration of ventilatory strategies in this population.
